# Different cultivation systems and foliar application of calcium nanoparticles affect the growth and physiological characteristics of pennyroyal (*Mentha pulegium* L.)

**DOI:** 10.1038/s41598-023-47855-6

**Published:** 2023-11-21

**Authors:** Hamid Reza Roosta, Arman Samadi, Mahdi Bikdeloo

**Affiliations:** Department of Horticultural Sciences, Faculty of Agriculture and Natural Resources, Arak University, Arak, 38156-8-8349 Iran

**Keywords:** Light responses, Plant physiology

## Abstract

The aim of this study was to investigate the impact of different cultivation systems (soil cultivation, hydroponic cultivation in greenhouse conditions, and hydroponic vertical cultivation in plant factory under different LED lights) and foliar spraying of nano calcium carbonate on pennyroyal plants. Nano calcium carbonate was applied to the plants at a 7-day interval, three times, one month after planting. Results showed that the greenhouse cultivation system with calcium carbonate foliar spraying produced the highest amount of shoot and root fresh mass in plants. Additionally, foliar spraying of calcium carbonate increased internode length and leaf area in various cultivation systems. Comparing the effects of different light spectrums revealed that red light increased internode length while decreasing leaf length, leaf area, and plant carotenoids. Blue light, on the other hand, increased the leaf area and root length of the plants. The hydroponic greenhouse cultivation system produced plants with the highest levels of chlorophyll, carotenoids, and phenolic compounds. White light-treated plants had less iron and calcium than those exposed to other light spectrums. In conclusion, pennyroyal plants grown in greenhouses or fields had better growth than those grown in plant factories under different light spectrums. Furthermore, the calcium foliar application improved the physiological and biochemical properties of the plants in all the studied systems.

## Introduction

Medicinal plants are always considered resources with economic value. The mint family includes various species of medicinal plants that are grown commercially due to their high marketability. Due to the high economic and medicinal importance of the plants of this family, the producers always seek to use new techniques to increase the production of mints^1^. Pennyroyal is a perennial plant that is usually propagated by division plant, of course, it can be propagated through stem and root cuttings. One of the characteristics of this plant is its pleasant taste, which is used as a spice vegetable in addition to its medicinal uses.

The world population will reach 9.7 billion people by 2050. At that time, a large percentage of the world's land will be unusable for agriculture. As a result, food production must increase by 110%. For this reason, the world needs to develop and apply methods to improve and increase the output of agricultural systems. Today, agricultural production systems need to change and develop the current systems^2^. In modern agriculture, proper fertilizer use is one of the necessities to improve crop yield and quality^3^. Improving the efficiency of nutrient use is required to protect the environment and increase crop productivity^4^. Nanotechnology is finding revolutionary applications to improve agricultural and food systems, especially for better crop production and food preservation^5^. Calcium is an essential element for plant growth and development and regulation of cellular processes^6^. Many studies have demonstrated that calcium is a main signaling molecule that plays a role in the defense response of plants against environmental stresses and plant growth^7^. According to the reports, the oregano plant has high requirements for calcium, the critical calcium concentration seems to be around 19 mg g^−1^ DW^8^. Thus, it may also be possible that the pennyroyal plant, as a member of the same family (Lamiaceae) needs high calcium, and foliar application of calcium can improve physiological characteristics and consequently increase yield. The most important advantages of foliar fertilization are to improve plant growth and crop quality, appropriately manage the nutritional status of plants, and regulate nutrient deficiencies. Soil application of calcium can increase crop yield as well as improve soil conditions, while foliar application has more advantages in correcting deficiency disorders of Ca^9^. It has been reported that calcium provided a larger number of leaves and biomass, thus Ca spraying resulted in increased growth of lettuce, showing that calcium is absorbed and distributed by the plants^10^. Increases in plant height, number of stems per plant, the concentration of chlorophyll, the yield of dry matter, and yield of essential oil were observed in oregano treated with 0.5% and 1% calcium chloride^8^. Foliar application of calcium to maize increased crop growth by increasing photosynthesis, water potential, stomatal conductivity, deposition of total soluble sugars, transpiration rate, and decreased hydrogen peroxide content that help the plant to thrive under drought stress conditions^11^.

Light causes changes in enzyme activities in the plant by affecting the production pathways of primary metabolites and thus affects the concentration of elements inside the plant^12^. Light is not only the source of energy for plant photosynthesis, but also an environmental signal that controls many aspects of plant growth and development. Light factors that affect plant growth and material metabolism include: light intensity, light quality, and photoperiod^13^. In recent years, to investigate the effect of light quality on nutrient absorption, monochromatic lights, and their different ratios have been widely studied^14^. Different qualities of light have different effects on plants, among which red light and blue light, which are related to the maximum absorption spectrum of photosynthetic pigments, have the most important effect on plants^13^. Further, UV added to white background lighting as well as monochromatic red light have been shown to increase essential oils contents in peppermint, however, monochromatic blue light as well as blue light added to white decreased the essential oil content^15^. The highest peppermint growth, as the most plant families, is obtained with red/blue light treatment^15^.

Little information is available about the pennyroyal growth and physiological characteristics in outdoor and indoor production systems especially plant factories with artificial lights. In this study, pennyroyal plants were grown in soil and soilless culture systems in the field, greenhouse, and plant factory and measured plant growth, pigments, total phenols, antioxidants, and Ca and Fe concentration parameters in response to different cultivation systems (including soil cultivation system, vertical hydroponic cultivation in the plant factory under different light spectrums, and hydroponic cultivation in the greenhouse) and nano calcium carbonate foliar nutrition.

## Materials and methods

To investigate the effect of different cultivation systems (soil, hydroponic under greenhouse conditions, and hydroponic in the plant factory under different LED lights) and the effect of nano calcium carbonate foliar spraying on the morphological and biochemical properties of the pennyroyal medicinal plant, a factorial experiment was conducted in the form of a completely randomized design with Six replications. In this experiment, the first experimental factor includes six cultivation systems (soil cultivation system, vertical hydroponic cultivation in the plant factory under red light, red/blue light, blue light, white light, and hydroponic cultivation in the greenhouse) and the second experimental factor includes two levels of foliar spraying of nano calcium carbonate (0 and 250 mg L^−1^).

### Cultivation method

**Figure 1:**
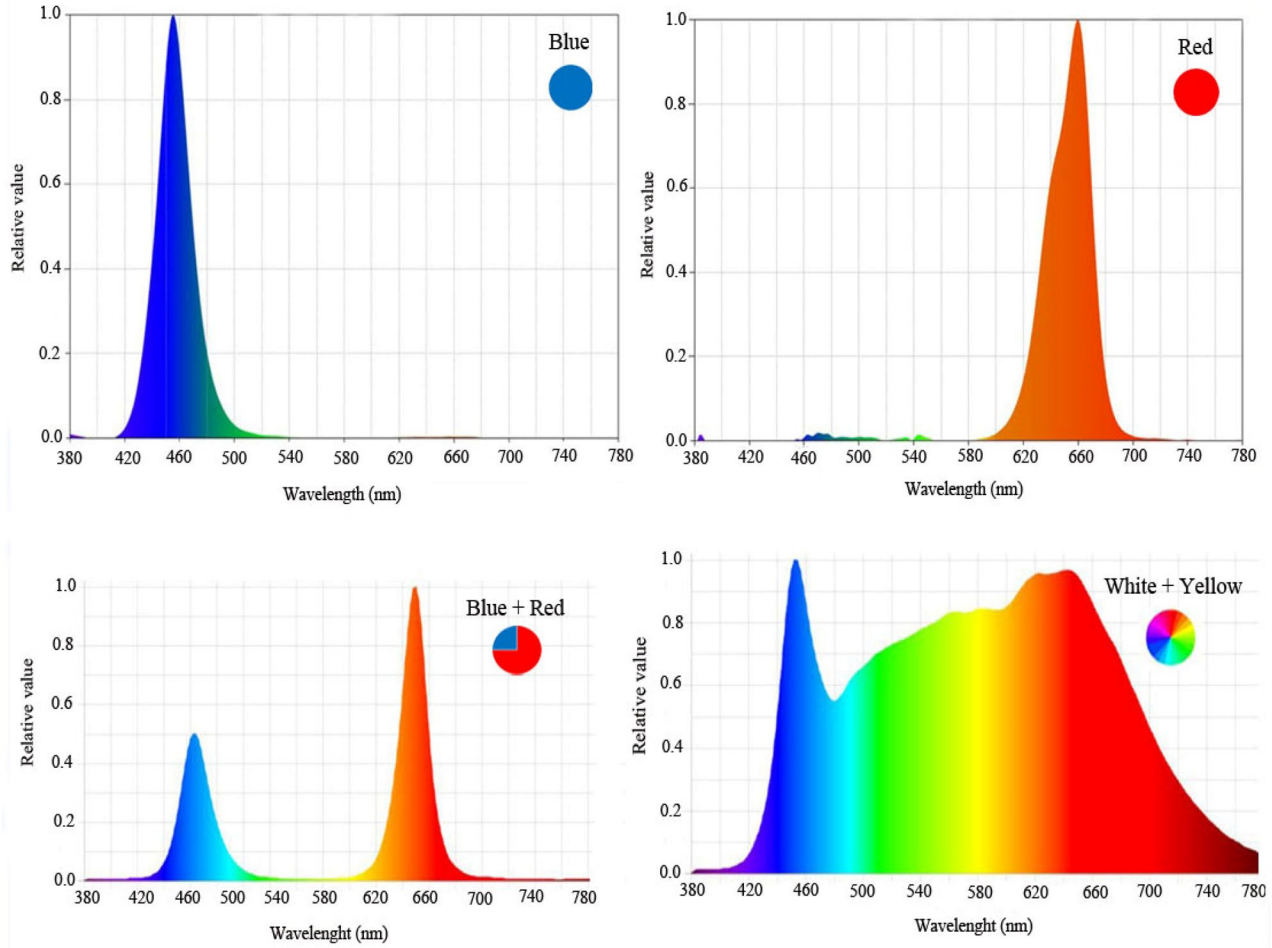
Relative distribution of different spectral LEDs (monochromatic blue, monochromatic red, red/blue (1:3) and white used during plant growth.

In this experiment, wild pennyroyal (*Mentha pulegium* L.) plants were collected from the Aqeel Abad region of Arak, Iran (geographical coordinates of the collection location are 49.6080669, 33.9788615) and were disinfected. Voucher specimens (128/1) were deposited in the herbarium of the Agriculture faculty of Arak University, Iran. The voucher specimen includes a planted collection and dry parts of plants in Arak University and Dr. Ahmad Reza Abbasifar identified it for our experiment. To perform disinfection, the roots of plants were first washed with water and disinfected with fungicide, then to obtain plants of the same size, all plants were first cultivated in perlite medium, and after two weeks, plants of the same size and shape were selected and planted in the different systems. To grow pennyroyal plants under farm conditions, first, fully decomposed manure was given to a land with an area of 12 m^2^, and then the land was plowed in the spring, and in June, pennyroyal plants were cultivated. The land was divided into three plots, 6 pennyroyal plants were planted on each plot, and irrigation was done immediately after planting. In the greenhouse and the plant factory, the plants were cultivated in the hydroponic cultivation systems, with the difference that the plants grown in the greenhouse were under the environmental conditions of the greenhouse, but plants in the plant factory were grown under LED lights with different spectrums (red, blue, white and red/blue lights) (Fig. 1). The plants were grown in 5-L buckets and an air pump was used to provide aeration. Morgan nutrient solution (Table 1) with electrical conductivity of 2.1 dS m^−1^ and pH of 5.8 was used to fertigate pennyroyal plants^16^. At the end of the experiment, vegetative growth parameters were measured before the flowering stage, and then the plants were harvested to evaluate the physiological characteristics.

**Table 1:** Concentration of nutrients used in the nutrient solution of this experiment.

Macronutrients	Concentration (mg L^−1^)	Micronutrients	Concentration (mg L^−1^)
N	128	Fe	5
P	58	Mn	2
K	211	Zn	0.25
Ca	104	B	0.7
Mg	40	Cu	0.07
S	54	Mo	0.05

### Foliar spraying of nano calcium carbonate

To prepare a solution of 250 mg L^−1^ of calcium carbonate nanoparticles, 0.25 g of calcium carbonate nanoparticles from the American Shell company were weighed, then it was ground and two drops of Tween 20 were added to one-liter solution. Distilled water was added and Tween 20 dissolved in water with a magnetic stirrer, and then calcium carbonate nanoparticles powder was added to the solution, and after stirring the solution, it was ready for foliar application. One month after planting the pennyroyal plants, the target plants were sprayed with calcium carbonate nanoparticles at a concentration of 250 mg L^−1^ three times at 1-week intervals. The control plants were sprayed only with distilled water. The spraying with the solution continued until the drops of the solution flowed from the plant leaves. The spraying was done in the early morning manually.

### LED tubes and light treatments

**Figure 2:**
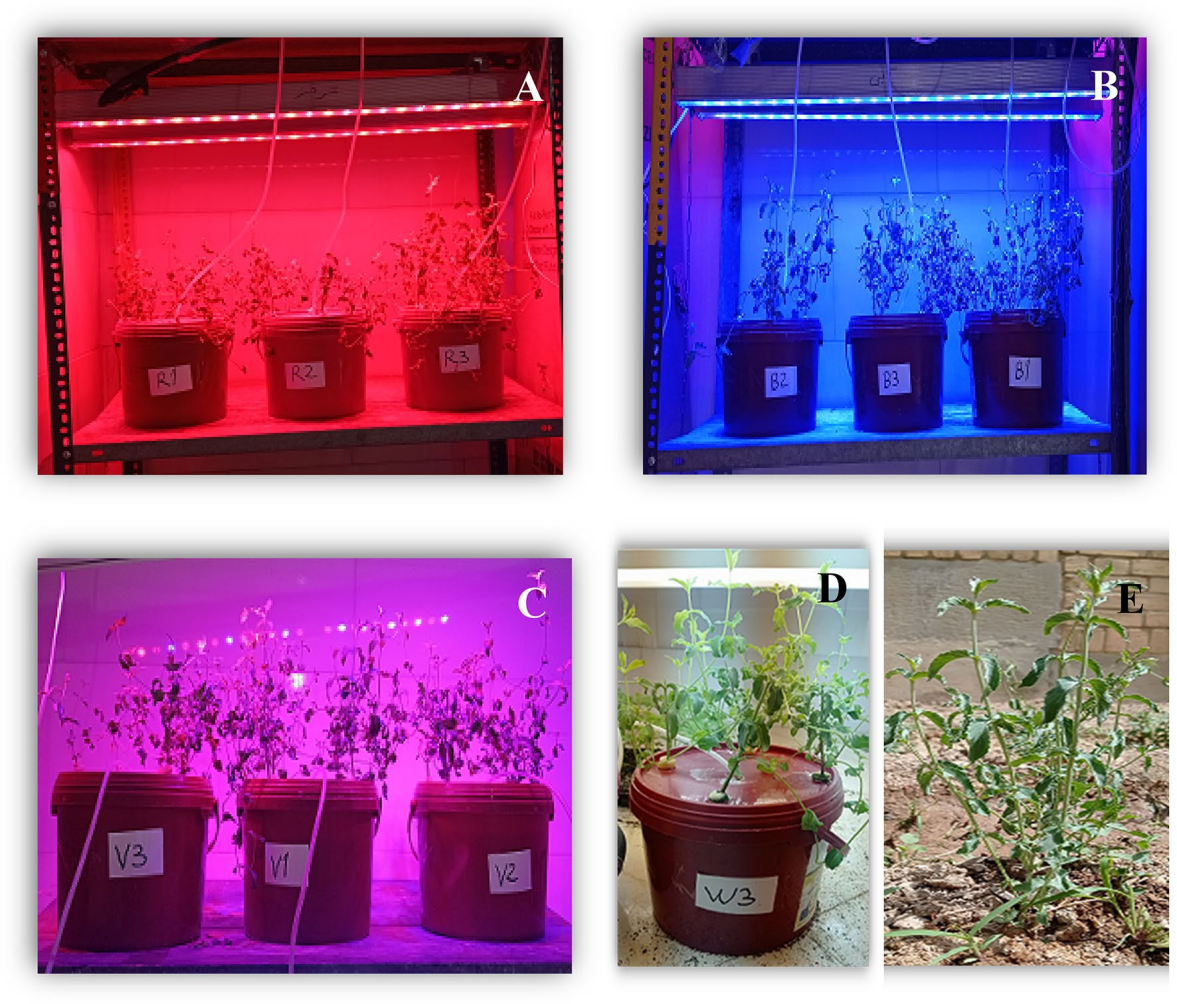
Different cultivation systems of the pennyroyal plants in the experiment. Plants that treated by different light spectra, (**A**) red, with a peak at 656 nm, (**B**) blue, with a peak at 450 nm, (**C**) red/blue 3:1, with a peak 656 nm, and (**D**) white (with a peak at 449 nm) were grown in the floated hydroponic system located in the plant factory. Plants in the greenhouse and (**E**) field conditions were grown in the ambient light.

In this study pennyroyal plants were grown under LED lamps with 24 × 3 W with 90% efficiency, 40 cm × 10 cm light coverage area, 600 mA ± 5% output current, and 50/60 Hz output frequency (Parto Roshd Novin Company, Iran) with different spectral ranges: [white (with a peak at 449 nm, blue (with a peak at 450 nm), red (with a peak at 656 nm), red/blue (3:1; R:B, with a peak 656 nm)] (Fig. 2). The photosynthetic photon flux density (PPFD) was 215 ± 5 μmol m^−2^ s^−1^ in all treatments. The photoperiod of 16/8 h (day/night) was maintained. The LED light systems were placed 30 cm above the plants.

### Vegetative growth parameters

Morphological traits including root fresh mass, shoot fresh mass, internode length, number of lateral branches, number of leaves, leaf area, root length, root dry mass, and shoot dry mass were measured. At the end of the study, the top of the plants was separated from the roots and the number of leaves and lateral branches were counted and the internode length, root length, and leaf length and width were determined. The fresh mass of shoots and roots was measured using a digital scale. After measuring the fresh mass of shoots and roots, to measure the dry mass, the samples were placed in an oven at a temperature of 65 °C for 72 h, and after this period, their dry mass was measured. To measure the leaf area, the samples were scanned using Dijimizer software, and the leaf area was determined.

### Leaf pigments

To measure the amount of chlorophyll and carotenoids, 0.25 g of frozen leaf sample was poured into a porcelain mortar, then it was crushed well using liquid nitrogen. Twenty ml of 80% acetone was added to the sample, then it was centrifuged at 4800 rpm for 20 min. Using a spectrophotometer, the amount of luminescent absorption of the samples was measured at 470, 647, and 663 nm. Finally, the amount of total chlorophyll and carotenoids was calculated according to Lichtenthaler^17^.

### Total phenols, flavonoids, antioxidants

To measure total phenol, the Folin-Ciocalteu colorimetric method was used. 0.2 g of dry plant leaves were ground with 10 mL of 80% methanol and placed in a shaker for 3 hours, after which it was centrifuged for 10 min at 10,000 rpm at 4 °C. Then 400 µL of the obtained extract was mixed with two mL of Folin Ciocaltio reagent (1:10) and after a few minutes, 1.6 mL of 7.5% sodium carbonate was added to it. After incubating for 30 min at room temperature and in the dark conditions, the absorbance of the resulting mixture at 765 nm wavelength was read by a spectrophotometer. The concentration of phenolic compounds in the samples was determined using the calibration curve prepared by gallic acid^18^.

To measure the total antioxidant and flavonoid contents, 0.5 g of dry and powdered leaf sample was weighed and poured into 10 ml of 80% methanol in a test tube, then the samples were placed in a shaker with 10,000 rpm for 24 h. After that, it was placed in an LC-04A centrifuge at 5000 rpm for 20 min, and then the supernatant solution was used to perform the analysis.

The amount of flavonoids in the samples was determined by the colorimetric method of aluminum chloride (AlCl3)^19^. For this purpose, 2 mL of the methanol extract of the samples was mixed with 2 mL of 2% aluminum chloride in a dark test tube. After keeping the samples at room temperature for 15 minutes, the absorbance of the samples was read at 415 nm.

For antioxidants measurement, 0.1 mL of the extract solution was mixed with 1 mL of the reagent (0.6 M sulfuric acid, 28 M sodium phosphate, and 4 M Ammonium molybdate) in a test tube and left for 1.5 h in the hot water bath at 95 °C. After this time, the samples were placed at room temperature for 30 min to cool down and a blue-colored complex was formed. Then, the absorbance of the solution was measured at a wavelength of 695 nm using the spectrophotometer (Specord 200 Plus, Analytik Jena). To obtain the antioxidant concentration, a standard solution of ascorbic acid (prepared on the same day of measurement) was used in concentrations of 0.03 to 1 mg L^−1^^20^.

### Calcium and iron

In this research, the analysis of calcium and iron contents in a leaf sample was performed using the ICP-OES (Analytic Jena Co., Germany). The total concentrations of Fe and Ca in plant shoots were determined according to standard methods following HNO_3_ digestion^21^. Before digestion, each sample was powdered and weighed and approximately 0.3 g of dry and powdered leaf samples, HNO_3_ (6 mL, concentrated) and H_2_O_2_ (2 mL, concentrated) were mixed into a beaker vessel. All samples were digested based on the hot plat method while a watch glass was placed on the vessel. Hydrogen peroxide was added to aid the digestion of the organic matrix (refer to Thermo Scientific AN 443662 and AN 4344620). A hot plate digestion system equipped with a stirrer and a temperature sensor was used for the digestion. The samples were heated based on the temperature programing as follows: a temperature of 50 °C for 1h, 150 °C for 1h, and then 200 °C for another 1 h to obtain brown color gas. To complete the removal of gas, digestion samples were kept for 15 min at static conditions. After digestion, each sample was transferred to a volumetric flask (25 mL). The flask was then made up to volume with ultrapure water before analysis by ICP-OES.

### Data analysis

Statistical analysis of the data was done using SAS software version 9/4, and drawing graphs and tables and displaying information was done using Excel software, and the comparison between the means was done using the Duncan test at a probability level of 5%.

### Statement of compliance

The authors confirm that permissions or licenses to collect *Mentha pulegium* L. plants were obtained from the Ministry of Agriculture-Jahad of I.R. Iran. In the all experiments on plants/plant parts in the present study, the use of plants complies with international, national and/or institutional guidelines.

## Results and discussion

### Plant growth

According to the results, the highest shoot fresh mass of plants was observed in the greenhouse cultivation system with calcium carbonate foliar spraying. The lowest shoot fresh mass was observed in the red/blue light treated plants without calcium carbonate foliar spraying, which was significantly increased with calcium nanoparticle foliar spraying (Table 2). The shoot fresh mass of the plants grown in the field was lower than that of the foliar-sprayed greenhouse plants, but it was higher than that of the plants grown in the plant factory. The foliar application of calcium nanochelate on the sweet basil plant has improved the yield compared to the control plants^22^. Calcium has an important role in the cell division process and cell wall formation, and consequently in plant growth and development. Foliar spraying of calcium lactate in lettuce plants in the field cultivation system under normal irrigation and drought stress conditions increased plant fresh mass and total yield^23^. In the current experiment, due to the high concentration of calcium in the field grown plants and no effect of calcium spray on the concentration of this element, foliar application of calcium nanoparticles had no significant effect on the shoot fresh mass of these plants. On the other hand, calcium spray increased the concentration of this element, and consequently shoot fresh mass in greenhouse-grown plants. Su et al.^24^ reported that red light has an inhibitory effect on plant height, plant fresh mass, and leaf area of cucumber seedlings. In our experiment, the low shoot fresh mass of the plants in plant factory might be due to insufficient light intensity, which may increase with the elevation of light intensity to higher levels. Though based on experiments conducted with diverse lighting technologies, light intensities ranging from 113 to 1200 μmol m^−2^ s^−1^ have been suggested for different mint developmental stages^25–27^.

**Table 2:** The interaction of different cultivation systems and nano-calcium carbonate foliar spraying on the vegetative growth parameters of the pennyroyal plants.

Foliar nano-Ca spray	Cultivation system	Shoot fresh mass (g plant^−1^)	Root fresh mass (g plant^−1^)	Shoot dry mass (g plant^−1^)	Root dry mass (g plant^−1^)	Internode length (cm)	Leaf no (no. plant^−1^)	Root length (cm)
No spray (control)	Red light	5.48ef	0.73d	0.62c	0.19d	4.97ab	135d	14.08ef
	Blue light	8.41d	1.14d	0.84c	0.16d	2.64g	213c	19.17cd
	Red/blue light	3.96f	0.58d	0.48c	0.16d	4.14de	208c	12.67f
	White light	6.59de	1.45d	0.78c	0.24d	3.20f	185c	15.83def
	Greenhouse	14.14c	5.10c	3.22b	0.70c	3.86e	211c	30.67a
	Field	17.52b	7.82b	5.94a	1.83a	5.33a	183c	20.67c
Nano-Ca spray	Red light	7.77de	0.82d	0.89c	0.20d	5.19ab	196c	13.17ef
	Blue light	8.38d	1.71d	1.04c	0.27d	2.66g	187c	18.00cd
	Red/blue light	6.77de	1.85d	0.69c	0.27d	3.89de	264b	16.50de
	White light	5.97def	1.50d	0.70c	0.13d	3.91de	193c	15.91def
	Greenhouse	32.92a	12.26a	5.43a	1.37b	4.39cd	407a	25.83b
	Field	18.28b	6.22c	5.34a	1.37b	4.72bc	192c	24.83b

Plants that treated by different light spectra (white, with a peak at 449 nm, blue, with a peak at 450 nm, red, with a peak at 656 nm and red/blue 3:1, with a peak 656 nm) were grown in the floated hydroponic system located in the plant factory. Plants in the greenhouse and field conditions were grown in the ambient light.

Mean separation was done by Duncan’s multiple range test and the same letter(s) in each column indicates non-significant difference at P ≤ 0.05.

The highest root fresh mass was also observed in the plants grown in the greenhouse cultivation system with calcium carbonate foliar spraying (Table 2). The root fresh mass in the plants grown in the plant factory under different spectrums was the lowest with no significant difference between foliar calcium carbonate sprayed plants and non-sprayed plants. The root fresh mass of the plants grown in the field was lower than that of the foliar-sprayed greenhouse plants, but it was higher than that of the plants grown in the plant factory. Foliar application of calcium nanoparticles decreased the root fresh mass of the plants grown in the field. It has been reported that plant height and stem diameter in tomato plants increased significantly with red and blue/red lights compared to white light, but root growth decreased in red light^28^.

The highest internode length was observed in the field cultivation system without calcium carbonate foliar spraying, and the lowest internode length was observed in the plants grown in the blue light cultivation system. Among the plants grown in the vertical cultivation system under different light spectrums, the largest internode length was observed in plants under red light (Table 2). The blue wavelength decreases the length of the internodes of the plant, while receiving high infrared light by the photopigments leads to an increase in the length of the internodes. Blue light influences the photoreceptor cryptochromes, which are responsible for controlling the height of the plant, causing to decrease in the length of internodes and stem length^29^. In the vertical cultivation systems, low blue/green light ratios induced such excessive stem and branch elongations, that peppermint cultivation remained constrained to the vegetative phase. In contrast, red/blue with its high blue light ratio induced compact growth (appropriate for prolonged space-limited cultivation of peppermint)^15^.

Lin et al.^30^ reported that internode length, shoot and root fresh mass, and plant height were higher in purple basil plants under red LED light than plants under blue and red/blue light.

**Figure 3:**
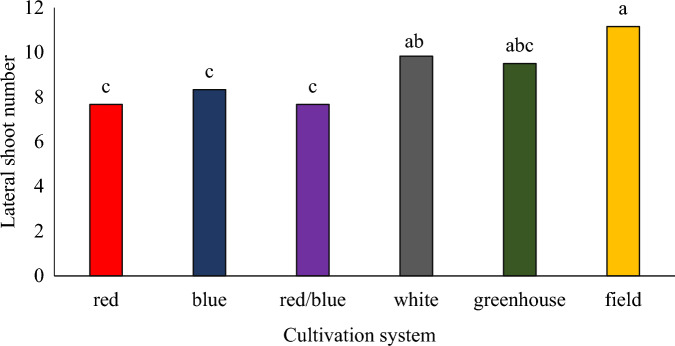
The effect of different cultivation systems on the number of lateral branches of pennyroyal plant. Plants that treated by different light spectra (white, with a peak at 449 nm, blue, with a peak at 450 nm, red, with a peak at 656 nm and red/blue 3:1, with a peak 656 nm) were grown in the floated hydroponic system located in the plant factory. Plants in the greenhouse and field conditions were grown in the ambient light.

The plants grown in the vertical cultivation system under red, blue, and blue/red light did not differ significantly in terms of the number of lateral branches, although in the vertical cultivation system, the white light spectrum increased the number of lateral branches compared to other spectra. The highest number of lateral branches was observed in the plants grown in the field cultivation system (Fig. 3). Girón González et al.^31^ reported that light quality is a factor that can affect the branching pattern of plants. As a result, reducing the ratio of red to far-red light in a dense plant population increases terminal dominance and ultimately reduces branching in plants. Ahmadi et al.^32^ reported that the highest number of leaves and lateral branches in the lemongrass plant were observed under blue/red light. Although, in the current experiment calcium spray has no effect on the number of lateral branches, foliar calcium application in oregano was shown to reach the meristem and favored growth with an increase in the number of stems per plant^8^.

Results showed that nano calcium foliar application increased the number of leaves of pennyroyal plants in vertical cultivation systems with red and blue/red light spectrums and greenhouse hydroponic cultivation (Table 2). The highest number of leaves was observed in the plants grown in the greenhouse and sprayed with nano calcium, however, in the vertical cultivation system, the blue/red light spectrum combined with nano calcium foliar application increased the number of leaves compared to other spectra. The lowest number of leaves was observed in the vertical cultivation system and red light spectrum without nano calcium foliar spraying. Improvement and performance in plants by foliar application of calcium has already been confirmed. It has been reported that nano calcium carbonate increases the number of leaves of the golden rain tree (*Koelreuteria panicuata*), which is similar to the result of the present study^33^. The application of calcium compounds at all light levels significantly increased the number of lettuce leaves compared to the control^34^. Ahmadi et al.^32^ reported that the highest number of leaves in the lemongrass plant were observed under blue/red light.

**Figure 4:**
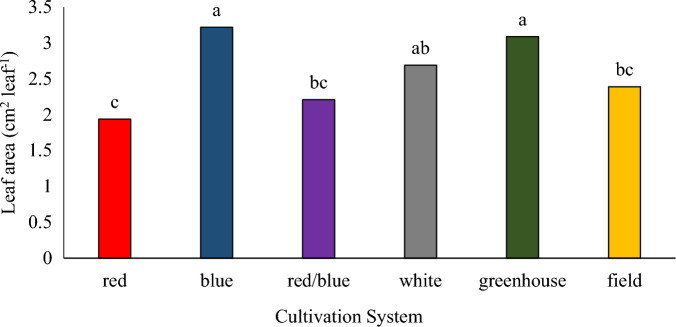
The effect of different cultivation systems on the leaf surface of pennyroyal plant. Plants that treated by different light spectra (white, with a peak at 449 nm, blue, with a peak at 450 nm, red, with a peak at 656 nm and red/blue 3:1, with a peak 656 nm) were grown in the floated hydroponic system located in the plant factory. Plants in the greenhouse and field conditions were grown in the ambient light.

**Figure 5:**
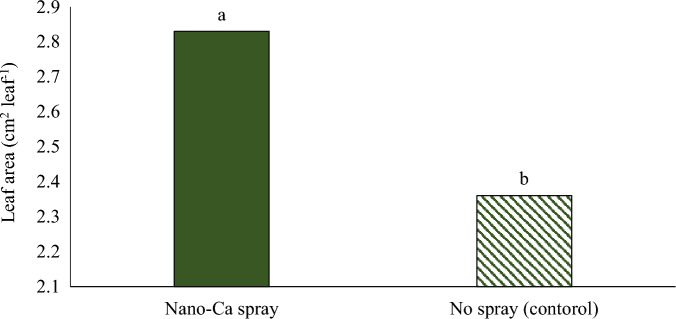
The effect of foliar application of nano calcium carbonate on the leaf area of pennyroyal plants.

The results showed that the leaf area of pennyroyal plants was the highest in the greenhouse cultivation system and vertical cultivation system under blue light (Fig. 4). The plants cultivated in the vertical cultivation system under red light had less leaf area compared to other plants. Foliar spraying of plants with nano calcium carbonate caused a significant increase in the leaf area compared to the control (Fig. 5). Jensen et al.^35^ reported that blue light increases the leaf area in lettuce plants. It has been suggested in a report that soybean seedlings grown under different combinations of red/blue light had more leaf area than seedlings grown under red light, and the results of this study are consistent with the results obtained from previous studies^36^. Foliar spraying of nano calcium carbonate improved growth indicators including leaf area and increased seed yield in basil plants^22^.

The highest amount of root length in different cultivation systems was observed in the plants cultivated in the greenhouse hydroponic system. In the vertical cultivation system, among different light spectrums, the highest and lowest amount of root length was observed in the blue and blue/red light spectrum, respectively (Table 2). Spaninks et al.^37^ suggest that the balance between shoot and root elongation in Arabidopsis and tomato seedlings can be controlled by the Red/Blue light ratio in the spectrum. Lim and Eom^38^ reported that blue light hastens root formation in basil cuttings. Blue light upregulates auxin signaling IAA accumulation induces both cell division and expansion and thereby promotes root initiation and elongation^39^.

The highest amount of shoot dry mass was observed in the cultivation system of the field and greenhouse. There was no significant difference in terms of shoot dry mass in plants grown under red, blue, blue/red, and white light in the presence of calcium carbonate or without calcium carbonate (Table 2). Although nano calcium foliar application in the field plants did not have a significant effect on the shoot dry mass of the plants, it increased this trait in the greenhouse grown plants. An increase in the dry mass of rice plants has been reported as a result of treatment with calcium phosphate nanoparticles^40^. Bian et al.^41^ reported that the highest dry and fresh mass, and leaf area of tomato plants were observed in the combination of red and blue LED lights.

In the field cultivation system, the plants without foliar application of nano calcium carbonate significantly had the highest root dry mass, also there was a significant difference in terms of root dry mass in the plants grown in the vertical cultivation system under red, blue, blue/red and white light with or without calcium carbonate application. The lowest amount of root dry mass was observed in the vertical cultivation system and white light spectrum with calcium carbonate foliar application (Table 2). The results of the research on peppermint plants under different LED light treatments showed that the fresh and dry mass of the plants did not differ significantly from each other^15^, which was consistent with the results of the current research.

### Leaf pigments

**Figure 6:**
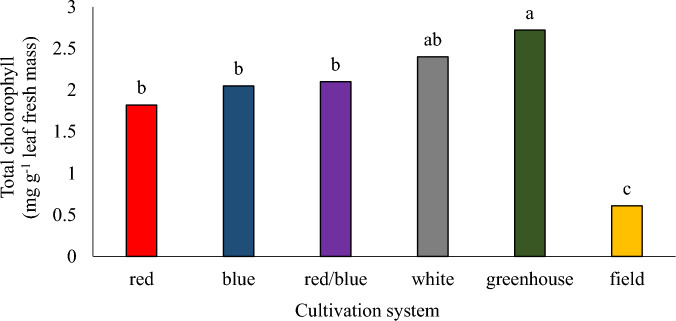
The effect of different cultivation systems on the total chlorophyll of pennyroyal plants. Plants that treated by different light spectra (white, with a peak at 449 nm, blue, with a peak at 450 nm, red, with a peak at 656 nm and red/blue 3:1, with a peak 656 nm) were grown in the floated hydroponic system located in the plant factory. Plants in the greenhouse and field conditions were grown in the ambient light.

The highest and lowest amount of total chlorophyll was found in plants grown in greenhouse and field hydroponic cultivation systems, respectively, and there were no significant differences in terms of total chlorophyll among the plants in vertical cultivation systems under different spectrums (Fig. 6). Photosynthetic processes that take place in plants to produce carbohydrate, convert light energy into chemical energy, using different photoreceptors sensitive to light at specific wavelengths. The effects of light quality on plants are very complex. Plant species differ in their response to light quality, but red light and blue light generally have the greatest effect on the plant growth^42^. According to the research conducted by Ghahremani et al.^22^, they announced that the use of calcium nano-fertilizers increased chlorophyll in basil plants. Ahmadi et al.^32^ stated that the amount of chlorophyll of lemongrass under fluorescent lamps and LED lamps was not different. Sooriyapathirana et al^43^. reported that the chlorophyll content, as indicated by the SPAD, was significantly higher in greenhouse grown amaranths plants compared to the field grown plants. Because of the closed environment and the design, the relative humidity is higher in greenhouse conditions than in the field. On the other hand, low humidity, soil pH limitation, and other possible stresses in the field condition can decrease chlorophyll concentration in field grown plants^43^.

**Figure 7:**
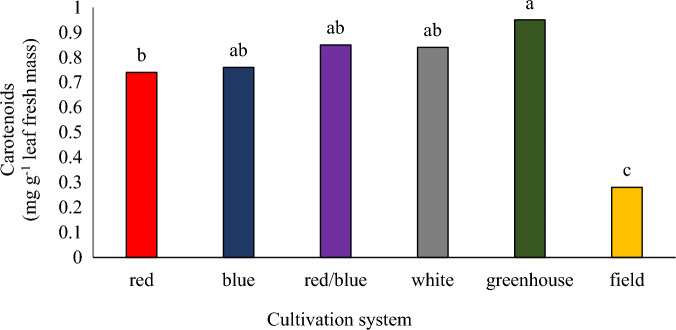
The effect of different cultivation systems on carotenoid content of pennyroyal plant. Plants that treated by different light spectra (white, with a peak at 449 nm, blue, with a peak at 450 nm, red, with a peak at 656 nm and red/blue 3:1, with a peak 656 nm) were grown in the floated hydroponic system located in the plant factory. Plants in the greenhouse and field conditions were grown in the ambient light.

The results of variance analysis of the data showed that the effect of nano calcium carbonate treatment and its interaction with culture systems was not significant on carotenoid levels (Data not shown). Also, there was no significant difference in carotenoid levels in cultivation systems under white, blue/red, and blue light. The plants grown in the greenhouse hydroponic cultivation system had the highest amount of carotenoids. Also, the lowest amount of carotenoid was observed in plants cultivated in the field (Fig. 7). Zhang et al.^44^ stated that blue light increases the amount of carotenoids in lettuce and citrus fruits. Chen et al.^13^ during a research on a lettuce variety under LED lights, reported the highest amount of carotenoids in red/blue and then blue light. The results of some studies have shown that the percentage of blue light has a direct effect on the accumulation of carotenoids in spinach, cabbage, basil, and pepper^29^.

### Total phenols

**Figure 8:**
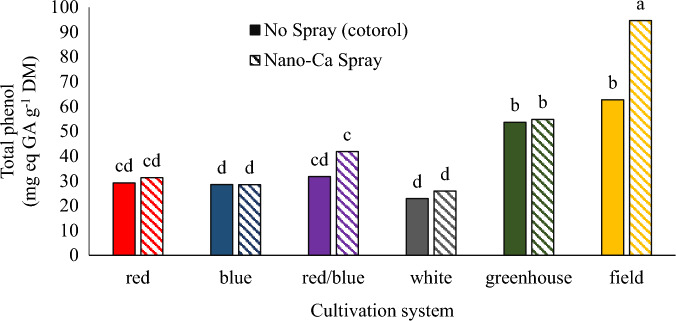
Interaction of different cultivation systems and calcium carbonate on total phenol of pennyroyal plants. Plants that treated by different light spectra (white, with a peak at 449 nm, blue, with a peak at 450 nm, red, with a peak at 656 nm and red/blue 3:1, with a peak 656 nm) were grown in the floated hydroponic system located in the plant factory. Plants in the greenhouse and field conditions were grown in the ambient light.

The results showed that the highest amount of total phenol was observed in the plants cultivated in the field cultivation system with calcium carbonate foliar spraying (Fig. 8). The amount of total phenol in the plants cultivated in the greenhouse hydroponic cultivation system was lower than the field-grown plants, but it was higher than plants grown in the plant factory. The environmental stresses have an important role in the secondary metabolite accumulation in plants^45^. The higher phenolic compounds in the field-grown plants could be due to the high stress conditions in the field compared to the plant factory and greenhouse. Different light spectrums and nano calcium carbonate foliar application did not affect the total phenol of the whole plant. Yi et al.^42^ stated that light can not only affect the primary metabolites of plants, but also affect the secondary metabolites of plants. Dou et al.^46^ stated that the use of red or blue supplementary light can affect the amount of phenolic compounds in some plant species, but the effects depend on the species and specific compounds. Bian et al.^41^ reported that continuous use of LED light for 24 h significantly increased the content of phenolic compounds and free radical scavenging capacity in lettuce leaves. In the experiments performed on a green basil cultivar, the illumination of the plant with a blue LED did not stimulate phenol synthesis^47^. Vafadar and Ehsanzadeh^48^ reported that calcium foliar application increased proline and phenolic compounds in lemongrass. Whereas, enhanced calcium supply to soybeans reduced the production of phenolic compounds and lignification due to low phenylalanine ammonia-lyase and peroxidases activities^49^. Thus, the calcium effect on phenolic compound concentration depends on plant species.

### Flavonoids

The results of the analysis of variance of data showed that the effects of different cultivation systems, calcium carbonate foliar application, and their interaction on the amount of flavonoids were not significant (Data not shown).

### Antioxidants

**Figure 9:**
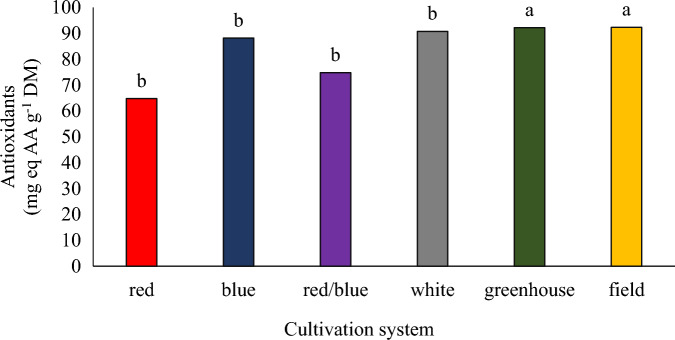
The effect of different cultivation systems on the antioxidants of pennyroyal plants. Plants that treated by different light spectra (white, with a peak at 449 nm, blue, with a peak at 450 nm, red, with a peak at 656 nm and red/blue 3:1, with a peak 656 nm) were grown in the floated hydroponic system located in the plant factory. Plants in the greenhouse and field conditions were grown in the ambient light.

There was no significant difference between the plants grown in the vertical system under red, blue, blue/red, and white lights. Also, there was no significant difference between the plants cultivated in the field cultivation system and greenhouse hydroponics in terms of the amount of antioxidants, but the antioxidants of these plants were higher than the plants grown in the plant factory (Fig. 9). Taulavuori et al.^50^ reported that the wavelength of light reflected to growing basil plants affects leaf size, essential oil, and the concentration of soluble phenols, some of which are antioxidants. Nguyen^51^ showed that there was no significant difference in antioxidant capacity between purple basil plants grown under blue and red light. In a study on Chinese kale, the antioxidant capacity of plants grown under blue light was higher than plants grown under red light^52^. In the controlled environments, the use of red and blue light was important to increase plant growth, greenness index, and antioxidant capacity in vegetables^29^. It is reported that light intensity (300 µmol m^−2^ s^−1^) would be an important factor in inducing antioxidant synthesis of basil plants^47^. In the current experiment, the light intensity in the plant factory was lower than 300 µmol m^−2^ s^−1^ (200 µmol m^−2^ s^−1^), which might be lower than the pennyroyal plant demand.

### Calcium concentration

**Figure 10:**
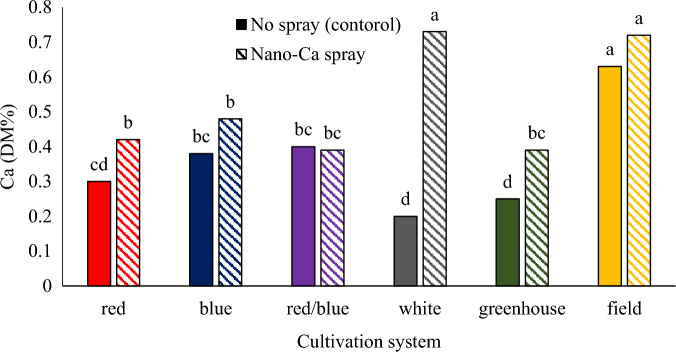
The interaction of different cultivation systems and calcium carbonate on the amount of calcium of pennyroyal plants. Plants that treated by different light spectra (white, with a peak at 449 nm, blue, with a peak at 450 nm, red, with a peak at 656 nm and red/blue 3:1, with a peak 656 nm) were grown in the floated hydroponic system located in the plant factory. Plants in the greenhouse and field conditions were grown in the ambient light.

Foliar spraying of calcium carbonate in different cultivation systems caused a significant increase in the concentration of calcium compared to control plants, so the highest concentration of calcium was observed in the field cultivation system and vertical cultivation system under white light with calcium carbonate foliar spraying. Also, the lowest concentration of calcium was observed in the plants grown in the vertical cultivation system under white light and the greenhouse hydroponic cultivation system without calcium carbonate foliar spraying (Fig. 10). Low calcium concentration in the plants grown in plant factory can be related to low transpiration of plants in this cultivation system. To determine the nutritional value of the plant, it is necessary to measure the amount of mineral elements. Measuring nutrients in plant tissue can be used as a tool to detect plant response to growth conditions as well as changes in growth and physiological indicators in plants^53^. Light causes changes in enzyme activities in the plant by influencing the production pathways of primary metabolites and thus affects the concentration of elements inside the plant^12^. In a research conducted on the coriander plant, the results showed that the amount of magnesium and calcium of the plant did not differ from each other under red, blue, and red/blue lights^51^. The amount of calcium in lettuce grown under blue light was significantly higher than in plants grown under red light^34^. In an experiment, the effect of calcium foliar application on the quality and quantity of cherry tomato fruit was investigated. The results showed that calcium foliar application led to an increase in vitamin C content and calcium content in cherry tomato fruit^54^.

### Fe concentration

**Figure 11:**
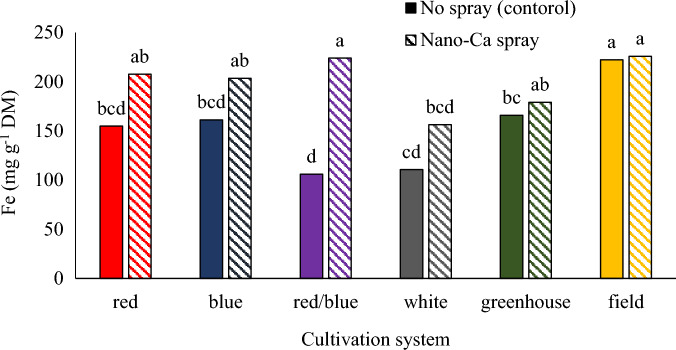
The interaction of different cultivation systems and calcium carbonate on the iron concentration in pennyroyal plants. Plants that treated by different light spectra (white, with a peak at 449 nm, blue, with a peak at 450 nm, red, with a peak at 656 nm and red/blue 3:1, with a peak 656 nm) were grown in the floated hydroponic system located in the plant factory. Plants in the greenhouse and field conditions were grown in the ambient light.

The highest concentration of iron was observed in the plants grown in the field system and the vertical system under blue/red light with calcium carbonate foliar spraying, but the lowest amount of iron was observed in the vertical cultivation system under blue/red light without calcium carbonate foliar spraying. Foliar spraying of calcium nanoparticles increased Fe concentration in plants grown in the plant factory and treated with red/blue light, significantly (Fig. 11). According to the results of Porter's research, the concentration of iron in plants cultivated in the field cultivation system was higher compared to the cultivation of plants in greenhouse environmental conditions and hydroponic cultivation system without foliar application of calcium carbonate. The high iron concentration in field conditions can be caused by more primary growth of the stem in the early growth, which leads to more root growth to obtain nutrients^53^.

## Conclusions

The results obtained from this study showed that the targeted use of calcium nano fertilizer and different conditions in the cultivation systems can be a step towards improving plant growth and its physiological characteristics. The results of the present study showed that the plants planted in a hydroponic greenhouse cultivation system had the highest amount of total chlorophyll and carotenoid, although calcium carbonate foliar application did not affect the amount of total chlorophyll and carotenoid. The plants planted in the greenhouse cultivation system with calcium carbonate foliar application showed the highest amount of total phenol. According to the results obtained in this study, it is possible to use the greenhouse cultivation system with calcium carbonate foliar spraying to increase the number of leaves, leaf area, root length, and shoot and root fresh mass of pennyroyal plants. The comparison of treatments related to different light spectrums showed that red light increased the internode length and root dry mass percentage, while it decreased the leaf length, leaf area, and plant carotenoids, while blue light increased the leaf area and root length in the pennyroyal plants. In the plant factory, pennyroyal plants treated with white light showed less iron and calcium content than other light spectrums. In general, it can be concluded that the growth of pennyroyal plants in the greenhouse and field systems was better than the cultivation systems in the plant factory in the presence of different light spectrums, and calcium foliar application improved the physiological and biochemical characteristics of the plants in all the studied systems.

## Data Availability

The data presented in this study are available on request from the corresponding authors. The data are not public.
